# Yeast as a tool to identify anti-aging compounds

**DOI:** 10.1093/femsyr/foy020

**Published:** 2018-03-04

**Authors:** Andreas Zimmermann, Sebastian Hofer, Tobias Pendl, Katharina Kainz, Frank Madeo, Didac Carmona-Gutierrez

**Affiliations:** 1 Institute of Molecular Biosciences, NAWI Graz, University of Graz, Graz, 8010, Austria; 2 BioTechMed Graz, Graz, 8010, Austria

**Keywords:** pharmacological screen, anti-aging, age-related disease, yeast, chemogenomics, drug discovery

## Abstract

In the search for interventions against aging and age-related diseases, biological screening platforms are indispensable tools to identify anti-aging compounds among large substance libraries. The budding yeast, *Saccharomyces cerevisiae*, has emerged as a powerful chemical and genetic screening platform, as it combines a rapid workflow with experimental amenability and the availability of a wide range of genetic mutant libraries. Given the amount of conserved genes and aging mechanisms between yeast and human, testing candidate anti-aging substances in yeast gene-deletion or overexpression collections, or *de novo* derived mutants, has proven highly successful in finding potential molecular targets. Yeast-based studies, for example, have led to the discovery of the polyphenol resveratrol and the natural polyamine spermidine as potential anti-aging agents. Here, we present strategies for pharmacological anti-aging screens in yeast, discuss common pitfalls and summarize studies that have used yeast for drug discovery and target identification.

## Teaser

The availability of various genetic mutant libraries combined with a high degree of mechanistic conservation relevant to aging make yeast a powerful screening platform for the identification and mechanistic study of novel anti-aging compounds.

**Abbreviations:**
RLS: Replicative lifespan
CLS: Chronological lifespan
FCG: Forward chemical genetics
RCG: Reverse chemical genetics
ROS: Reactive oxygen species
HTS: High-throughput screen
HIP: Haploinsufficiency profiling
HOP: Homozygous profiling
MSP: Multicopy suppression profiling
RMS: Random mutation suppression
CR: Caloric restriction

## INTRODUCTION

Aging describes the multifaceted decline of cellular and organismal function over time, and represents a major risk factor for the susceptibility to diseases. Indeed, there is a profound overlap between cellular pathways that influence aging and those linked to cancer, neurodegeneration and cardiovascular disorders as well as metabolic syndrome (de Cabo *et al.*^2014^). Hence, recent efforts have aimed at the identification of molecules that decelerate the aging process *per se* and thus may act as a preventive measure that collectively ameliorates age-related diseases. *Vice versa*, therapeutics for such diseases may also prolong overall healthspan or lifespan. In the search for anti-aging interventions, biological screening platforms are important tools for drug discovery. Cell-based assays are of particular relevance as they have several advantages compared to *in vitro* screens. First, they enable a drug to be studied in the cellular context, including the ability to pass cellular membranes and to withstand immediate export by multidrug transporters. Second, drug candidates that exhibit cytotoxicity by off-target effects, or a different response of the desired target *in vivo* upon ligand binding, can easily be ruled out by monitoring cell viability. Third, cellular platforms are highly compatible with unbiased phenotypical screens, as the desired phenotype (e.g. improved survival during aging) can be selected for, irrespective of the molecular target. The requirements for such screening platforms include a high degree of conservation, an economic workflow, and simple and reliable readouts. One of the organisms that meets these needs is the budding yeast (*Saccharomyces cerevisiae*), a unicellular fungus that is widely used as a model for human aging and age-related diseases. Its unparalleled genetic tractability combined with the availability of whole genome homozygous and heterozygous gene deletion collections as well as overexpression libraries, make *S. cerevisiae* a versatile toolbox for chemogenomic screens. Due to its short generation time (∼90 min) and modest culturing requirements, yeast can also be grown rapidly in high-throughput experimental setups.

In this minireview, we summarize strategies for yeast-based drug discovery and give examples for successfully employed pharmacological screens in yeast with a focus on aging and age-related diseases.

## YEAST AS A MODEL FOR HUMAN AGING

When selecting an experimental model for the identification of anti-aging compounds, a high degree of evolutionary conservation is crucial. In yeast, many pathways that are relevant for aging and disease in humans are well conserved, including nutrient signaling, cell cycle regulation, DNA repair mechanisms, mitochondrial homeostasis, lipostasis, protein folding and secretion, proteostasis, stress response, and regulated cell death (Longo and Fabrizio 2002; Tenreiro and Outeiro 2010; Eisenberg and Büttner 2014; Lasserre *et al.*^2015^; Janssens and Veenhoff 2016; Knorre *et al.*^2016^; Bilinski, Bylak and Zadrag-Tecza 2017; Postnikoff, Johnson and Tyler 2017; Carmona-Gutierrez *et al.*^2018^). About 90% of the ∼6000 yeast genes have already been characterized and approximately 30% of the yeast genome is conserved to humans, based on sequence similarity (Stefanini, De Filippo and Cavalieri 2013). Transgenetic studies have revealed, however, that sequence similarity is a poor predictor of orthology (i.e. functional homology). In fact, about half (in some pathways over 90%) of the essential yeast proteins can be replaced with their human orthologs, even though the respective sequence similarity ranges from over 90% to as little as 9% (Kachroo *et al.*^2015^). The growing awareness that pathways related to human senescence display a high degree of conservation in *S. cerevisiae* and other unicellular fungi has increasingly promoted the use of yeast to understand signaling pathways and identify molecular players involved in aging, as well as to unveil and/or test potential anti-aging interventions. Indeed, yeast-aging phenotypes are surprisingly similar to human post-mitotic cellular aging (Longo and Fabrizio 2012). In the presence of sufficient nutrients, yeast cultures grow exponentially by asymmetric budding of daughter cells from mother cells. During cell division, mother cells retain damaged cellular material that accumulates over time, ensuring maximal health of their offspring. Eventually (after 20–25 cell divisions), the mother cells die and release their cellular material into the environment (Steinkraus, Kaeberlein and Kennedy 2008; Longo *et al.*^2012^). Upon nutrient scarcity (e.g. in nutrient-limited batch cultures) yeast enter a stationary phase and stop dividing. Depending on the culture conditions and strain, yeast lifespan in this post-mitotic phase ranges from a few days up to several weeks. This comparatively short lifespan is particularly convenient in large-scale aging studies, which in other typically used aging model organisms can take between 20 days (nematodes; Riddle *et al.*^1997^), over ∼3 months (fruit flies; Linford *et al.*^2013^) and up to ∼3 years (mice; Flurkey, Currer and Harrison *2007*).

The two stages of yeast aging, mitotic and post-mitotic aging, can be monitored using two different models, replicative lifespan (RLS), and chronological lifespan (CLS), respectively. RLS represents the number of cell divisions a mother cell can undergo before death and is a model for proliferating cells (e.g. undifferentiated stem cells) (Sinclair 2013). As budded daughter cells have to be separated from the mother cells, RLS methods are usually time-consuming and difficult to adapt to high-throughput approaches.

CLS models aging in post-mitotic/differentiated cells and is assessed by monitoring cell survival in stationary batch cultures after the diauxic shift. CLS is compatible with several high-throughput techniques such as flow cytometry (Carmona-Gutierrez *et al.*^2010^), outgrowth (Murakami *et al.*^2008^) or spotting assays (Teng and Hardwick 2013). It should be noted, though, that the batch culture aspect makes this model sensitive to growth behavior and the accumulation of degradation products (especially acetic acid) in the medium, which might confound the interpretation of aging phenotypes. Thus, growth rates and media pH should always be controlled, at least when validating results from high-throughput experiments. Recently, continuous flow cultures such as retentostat cultures (Boender *et al.*^2011^), where the cellular biomass is retained in the growth chamber by filtration and cells stop dividing, have gained attention as CLS models since they combine post-mitotic aging with constant nutrient supply, thus eliminating the disadvantages of batch cultures. Although these culturing methods must still be trimmed to fit high-throughput demands, they are attractive tools in the validation chain of batch culture-based screens.

**Figure 1.**
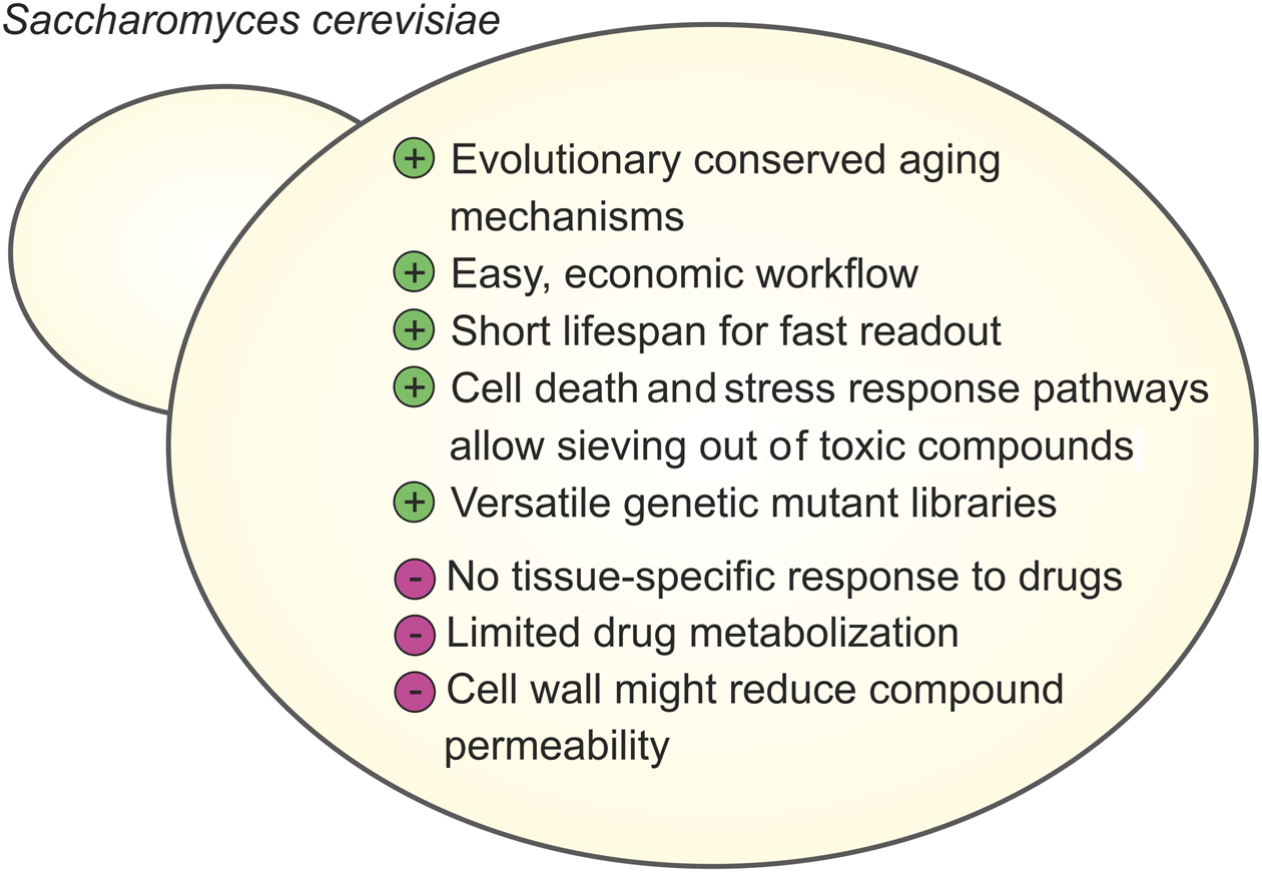
**Advantages and limitations of yeast for anti-aging drug discovery.** The budding yeast, *S. cerevisiae*, offers a genetically well-defined cellular environment paired with a fast and economic experimental workflow, including a variety of mutant libraries for drug target identification. Due to its unicellular nature, studying tissue-specific responses might be limited. Other constraints are limited drug metabolization and suboptimal permeability of the fungal cell wall to xenobiotics. See the main text for further details.

During yeast aging, typical age-associated phenotypical markers emerge, such as the accumulation of reactive oxygen species (ROS), the buildup of damaged organelles and proteins, DNA fragmentation, loss of membrane integrity and the increase of apoptotic/necrotic cell populations (Carmona-Gutierrez *et al.*^2010^; Janssens and Veenhoff 2016; Carmona-Gutierrez *et al.*^2018^) which bears many similarities to hallmarks of human aging (López-Otín *et al.*^2013^). To some extent, yeast might even be better suited to monitor aging on a cellular level other than human cell cultures because (i) due to the smaller size and growth in suspension of yeast cells, a larger number of cells can be monitored in a given culturing platform, especially compared to adherent human cell lines, which usually grow in monolayers (Montague *et al.*^2014^); (ii) in contrast to mammalian cell culture, where cells are extracted from their physiological environment within a heterogeneous tissue and thus only represent an isolated entity from a multicellular organism, yeast cell cultures represent an *in vivo* situation. There is even evidence for differentiation-like behavior of yeast cells when forming colonies on solid media (Cáp *et al.*^2012^), and some studies suggest a degree of crosstalk between individual cells (Herker *et al.*^2004^; Palková, Wilkinson and Váchová 2014). In terms of pharmacological screens, this may offer the possibility of using yeast as a rudimentary archetype for tissue microenvironments, although studying tissue-specific responses to drugs, including drug metabolization or prodrug activation, might exceed the power of the model (Resnick and Cox 2000). Consequently, while many intracellular mechanisms of aging regulation are well-conserved in yeast, extrinsic, intercellular factors, such as insulin-like growth factor (IGF-1) levels or inflammation, which influence aging in multicellular organisms (Franceschi *et al.*^2007^; Fontana, Vinciguerra and Longo 2012; Bartke 2017), cannot be investigated adequately in yeast. In addition, the fungal cell wall represents a barrier to some compounds, which, however, can be weakened genetically (see the chapter on ‘Pharmacokinetics and off-target effects in yeast’). Nevertheless, the easy handling and well-defined, highly conserved cellular environment have predestined yeast as a tool for drug discovery (Figure 1).

Besides *S. cerevisae*, other yeast species are used as aging models as well, in particular the fission yeast *Schizosaccharomyces pombe*. In fact, some cellular components that are poorly conserved between *S. cerevisiae* and mammals, such as the mRNA splicing machinery and nuclear structural proteins, are well-conserved in *S. pombe* (Roux *et al.*^2010^). Fission yeast is also preferably used to study mitochondrial inheritance, since this process is similarly regulated in human cells (Lin and Austriaco 2014). In addition, *S. pombe* has been used in pharmacological screens for anti-cancer agents (Satoh *et al.*^2017^) as well as genome wide screens to unravel the genetic network of drug-induced ROS accumulation (Hagihara *et al.*^2017^).

## PHARMACOLOGICAL SCREENING STRATEGIES

**Figure 2.**
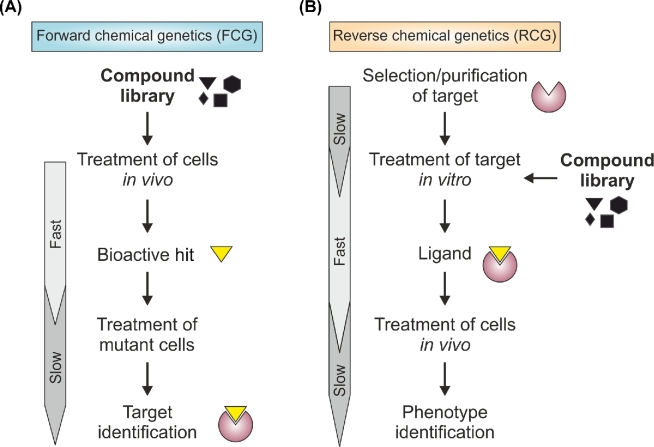
**Screening strategies for chemogenomic drug discovery. (A)** Forward chemical genetics (FCG) start with a compound library, which is administered to wild type cells or engineered yeast cells expressing a protein of interest. Hits are selected based on a phenotypic readout, ending up with bioactive compounds. Targets can be identified by treating genetic libraries (loss-of-function or gain-of-function mutants) with the identified hits and reversal of the phenotype as a readout. **(B)** Reverse chemical genetics (RCG) start with the selection of a target of interest, which is treated with a compound library *in vitro*, using ligand binding or enzymatic activity as readouts. Wild type cells or genetic libraries are treated with the selected hits to identify a phenotype and/or target.

In the search for novel bioactive compounds, there are two experimental approaches: (i) forward chemical genetics (FCG) or phenotypic screens, and (ii) reverse chemical genetics (RCG) or target-based screens (Figure 2). In FCG screens, cells are treated with substance libraries and hits are selected based on a phenotype (e.g. cell survival). When combined with libraries of different yeast mutants (e.g. gene deletion or overexpression strains), this strategy facilitates rapid identification of potential targets of a lead compound. Untargeted anti-aging screens typically follow this paradigm since aging is generally regarded as a multifactorial phenomenon which can be influenced by numerous (and often uncharacterized) cellular pathways in parallel (López-Otín *et al.*^2013^). Thus, an unbiased, multifactorial readout such as survival during aging may yield bioactive compounds that act on different targets but have the same phenotypical outcome. FCG screens allow assessment of all substances that might alter a desired phenotype, while potentially effective candidates with poor pharmacokinetic properties or side-effects masking the desired effect are sieved out automatically. Nevertheless, narrowing down a specific target of a bioactive drug remains challenging (Schenone *et al.*^2013^). RCG screens, on the other hand, circumvent this problem as the drug target is defined *a priori*, and chemical ligands are selected *in vitro* before testing their activity *in vivo*. Although there is still no comprehensive molecular characterization of the aging process, numerous potential targets for anti-aging drugs have been identified in genomic screens. For example, a large-scale screen with a collection of yeast deletion strains has indicated a lifespan-shortening role of purine biosynthesis (Matecic *et al.*^2010^). Given a feasible readout (e.g. activity measurements of the enzymes involved in the pathway), an RCG screen could help to identify purine biosynthesis modulators that, in turn, affect lifespan. However, a common disadvantage of RCG screens is that results from *in vitro* experiments, which usually use highly purified targets of interest isolated from their physiological environment, are often not transferable to the situation in a cellular context. Moreover, substances that act highly specifically *in vitro* might still exert significant side-effects *in vivo* that interfere with the desired phenotypical outcome. Therefore, hybrid setups, or *in situ* RCG screens, where the activity or downstream effects (e.g. in the case of a toxic transgene) of the target of interest is tested in genetically engineered yeast strains rather than *in vitro*, allow the advantages of combining both approaches.

## YEAST AS A CHEMOGENOMIC SCREENING PLATFORM

Limiting factors in the direct applicability of RCG and FCG screens in animals and human cell culture are the corresponding time, resource and experimental constrictions. In addition, animal models are less suited for FCG screens, because the complexity of genetic engineering impedes high-throughput testing of genetic mutants once a lead substance has been identified. Yeast, as an established model for a plethora of physiological and disease-relevant settings, offers a technical addendum to extensively exploit the quantitative and qualitative potential of such screens. One of the main advantages of yeast is the availability of a variety of strain libraries with loss-of-function or gain-of-function mutations of almost all genomic open reading frames (ORFs). Upon identification of an anti-aging compound candidate, such genetic libraries can help to identify drug targets without having to fundamentally change experimental setups.

The yeast knock-out collection (available via the European *Saccharomyces cerevisiae* Archive for Functional Analysis, www.euroscarf.de; or GE Dharmacon, dharmacon.gelifesciences.com) comprises ∼5100 strains in haploid genome background with deletions of non-essential genes (∼80% of all yeast genes). Essential genes can be studied in heterozygous diploid deletion strain libraries, or temperature-sensitive conditional mutants. The deletion strains are barcoded (unique DNA identifier for each strain), which enables enrichment analyses from a pooled mixture of strains via DNA barcode microarrays or barcode sequencing. Under the premise that in such a mixture of strains long-lived mutants will be enriched and short-lived mutants depleted over time, the knockout collection has successfully been employed in screens for genetic determinants of aging (Powers *et al.*^2006^; Murakami *et al.*^2008^; Matecic *et al.*^2010^). In a chemical screening context, drug-resistant mutants (when the compound inhibits growth) are enriched, while drug-sensitive strains show impaired growth compared to the untreated control and become underrepresented over time. This approach is even suitable for parallel identification of bioactive compounds and their respective targets. It is also termed drug-induced haploinsufficiency profiling (HIP) when working with heterozygous diploid strains, or homozygous profiling (HOP) when working with homozygous/haploid deletion strains. In contrast to HOP assays, where the potential target is deleted, HIP is based on a direct interaction of a chemical with its target. Both strategies may reveal mechanistic insights into disease-relevant drugs (Sun and Zhou 2016); however, they are highly sensitive to growth behavior *per se* and might lead to a high number of false hits. Indeed, in a HOP study which aimed at characterizing the influence of single amino acid substitutions in histones H3 and H4 on replicative aging, only 36% of the mutants identified in the initial DNA barcode enrichment screen could be validated in the subsequent low-throughput experiments (Sen *et al.*^2015^). As an alternative to enrichment assays, the knockout collection strains can also be tested independently in parallel using cell arrays and automated micorarraying devices. By pinning small amounts of liquid yeast cultures onto agar plates containing the TORC1 inhibitor rapamycin, and scanning for mutants that were able to form colonies, this procedure has been used to gain new insight into the rapamycin-responsive cellular network (Xie *et al.*^2005^). A considerable restriction is that the substance of interest has to be introduced into the agar plates; hence, the required amount of compound is several orders of magnitude higher than for setups in liquid culture. Moreover, this technique only works when the compound causes some degree of growth inhibition. Nevertheless, when working with compounds that prolong lifespan but do not inhibit growth, modified applications of such arrays may still be useful (see the chapter on ‘High-throughput methods to monitor yeast aging’). It should be noted that the yeast knockout collection does harbor a substantial amount of secondary mutations which might interfere with the chosen readout (Teng *et al.*^2013^). Thus, it is imperative to validate hits from such libraries by (i) complementation assays (e.g. by plasmid-based expression of the deleted gene) and/or (ii) recapitulation of the phenotype in *de novo* created deletion strains, ideally in different strain backgrounds.

The yeast ORF library (GE Dharmacon) offers over 4900 strains with galactose-inducible overexpression of single yeast genes which can be used for multicopy suppression profiling (MSP). The rationale of MSP is that a larger number of target proteins can increase the efficient dose of a drug that impairs growth. Again, this assay relies on differences in growth, which might be difficult to screen for in aging setups, especially when a drug does not cause an apparent growth phenotype. Nevertheless, such approaches confirmed a number of drug-target interactions, including rapamycin-Tor1p, tunicamycin-Alg7p and fluconazole-Erg11p (Giaever *et al.*^2004^; Hoon *et al.*^2008^).

Random mutation suppression (RMS) relies on spontaneous induced mutations of the genome, which suppress the effect of drug treatment. Random mutation libraries derived from transposon mutagenesis can easily be generated and used to identify loci linked to drug-resistance (Kumar 2016). Given the decreasing costs for whole genome sequencing, screens for spontaneous resistance by single nucleotide polymorphisms (SNPs) have become economically justifiable, simply by treating a large number of wild type cells and selecting for clones that are still able to form colonies in the presence of the compound. The genome of the resistant clone(s) can be sequenced to unravel the mutated locus. In fact, this rationale was followed to link a gain-of-function mutation in the peroxiredoxin Tsa1p to oxidative stress resistance after chemical mutagenesis (Timmermann *et al.*^2010^), and has also been used to identify genetic loci associated with drug treatment (Tardiff *et al.*^2013^).

In order to investigate specific pathways for *in situ* RCG screens, yeast can be genetically manipulated to facilitate high-throughput readouts. For example, target of rapamycin (TOR)-pathway activity can be monitored in strains carrying TOR-responsive promoters fused to fluorescent proteins and in quantification of the promoter activities by flow cytometry. Indeed, this method was used to identify a novel TORC1 inhibitor among a library of ∼320 000 compounds (Chen *et al.*^2012^). Of note, even though several pathway activities correlate with aging (e.g. mTOR signaling in neurons (Yang *et al.*^2012^), or the DNA damage response signaling routes p16INK4a/Rb (Ressler *et al.*^2006^) and p19ARF/p53 (Krishnamurthy *et al.*^2004^: 4)), there is no single signaling cascade that governs the aging process, or is specifically activated/deactivated during aging. There are several transcriptional pathways that have been linked with longevity, including several epigenetic modifications (Fahrenkrog 2015) such as silencing by the histone demethylase Rph1p (Schroeder, Raimundo and Shadel 2013), or the activation of stress-resistance transcription factors Msn2p/Msn4p (Fabrizio 2001) and Yap1p (Yiu *et al.*^2008^). However, at present, it is difficult to apply a reporter-based technique to unbiased screens for anti-aging compounds, simply because a comprehensive, stringent transcriptional response to aging that could be harnessed to identify compounds, which alter the response globally, has yet to be unraveled.

As an alternative to reporter-based screens, simple genetic ‘tricks’ may be used to amplify the phenotype after a given treatment, e.g. by linking the activity of a pathway to the expression of an essential gene. The difference in growth upon pathway modulation can be harnessed to embed pharmacological screens in pathway-specific approaches. For example, histone deacetylase (HDAC) Sir2p-mediated silencing of an engineered *URA3* gene (coding for a protein essential for growth in uracil-free media) at a telomeric region (strongly deacetylated by Sir2p) was used to identify novel Sir2p inhibitors by treating the cells with different compounds and to monitor growth in media lacking uracil (Bedalov *et al.*^2001^). A similar strategy served to search for Sir2p inhibitors, but this time scoring decreased growth in media containing 5-fluoroortic acid, which is converted to toxic 5-fluorouracil (5-FOA) by the product of the *URA3* gene (Grozinger *et al.*^2001^). In theory, variations of this approach can be used to screen for substances that activate Sir2p, e.g. by altering the genomic locus of the *URA3* insertion to a region which is silenced upon Sir2p activation (Li *et al.*^2013^) and monitoring growth in 5-FOA medium (thus, higher HDAC activity would reduce the levels of Ura3p and allow growth). Since Sir2p and its human homolog SIRT1 are widely implicated in the regulation of aging (see below), such approaches could yield attractive molecules for pharmacological anti-aging interventions. Alternatively, URA3-expression could also be put under control of a transcription factor of interest to identify activators/inhibitors of specific transcriptional signaling. However, this workflow is only compatible with semi-biased screens, as it limits the number of targets to the pathway of interest.

**Figure 3.**
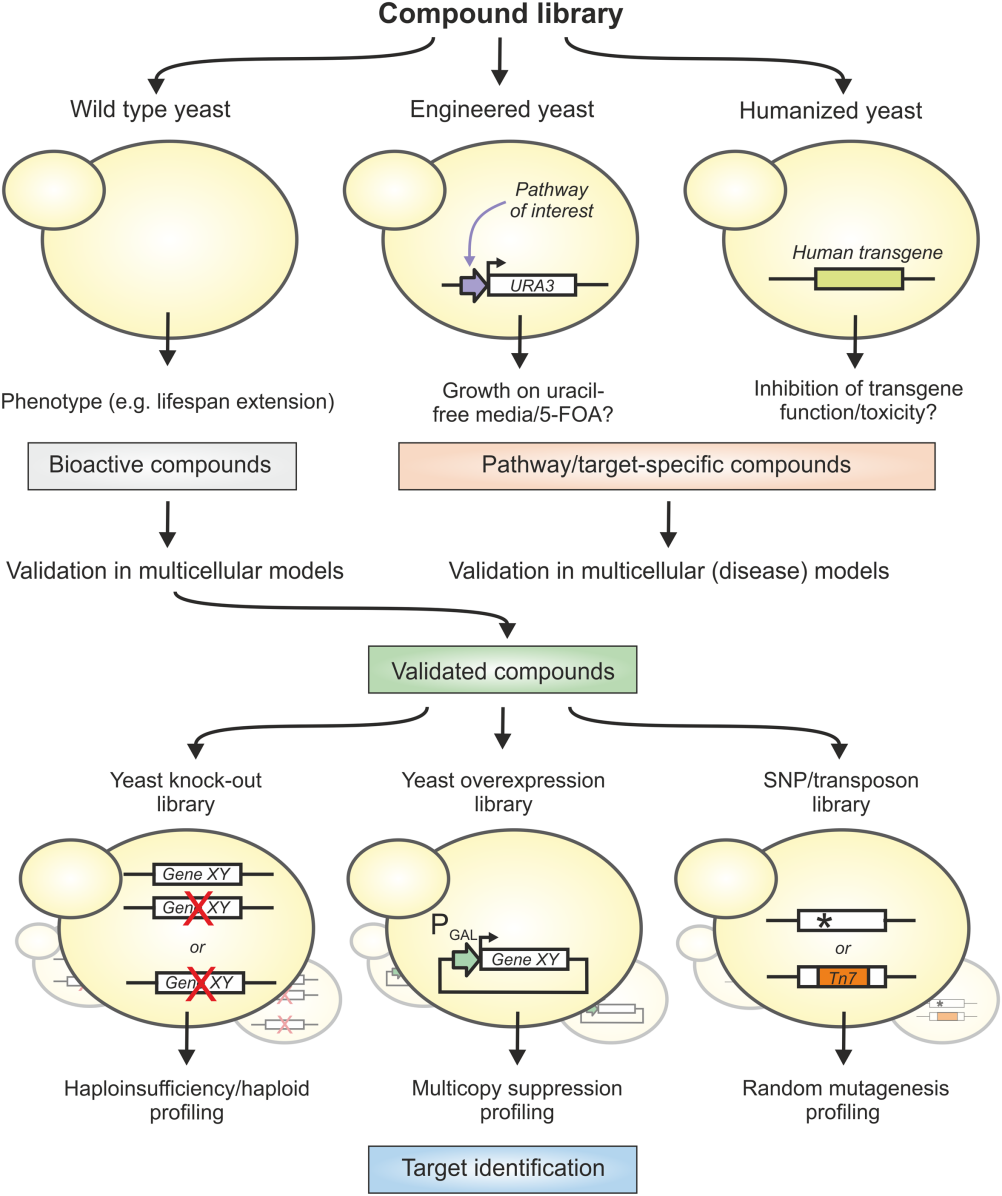
**Yeast as a pharmacological screening tool in forward chemical genetics.** Compound libraries can be tested in wild type yeast in an unbiased manner, or in semi-biased approaches in engineered/humanized yeast strains. To investigate specific pathways which do not result in an apparent phenotype, yeast can be manipulated to translate pathway activities into growth behavior. For instance, a promoter that is regulated by the pathway of interest can be linked to an essential gene (e.g. *URA3*). The growth on uracil-free or 5-fluoroorotic acid (5-FOA) containing media is an indirect measure for increased or decreased pathway activity, respectively. Humanized yeast, expressing human disease-relevant genes can be used to find compounds that specifically interfere with the function of the transgene. Compounds derived from unbiased screens should be validated in multicellular models before screening for potential targets in different yeast mutant libraries. Among others these libraries include heterozygous or haploid deletion as well as overexpression collections for different genes (*Gene XY*). In addition, *de novo* derived mutants by spontaneous (*) or transposon (*Tn7*) mutagenesis can be used to identify loci associated with the compound's mechanism of action.

A typical (FCG) yeast compound screening procedure has three phases: (i) an initial high-throughput compound screen using wild type yeast or a strain, specifically designed for the desired phenotype, for example, activation of a pathway of interest (using reporter- or growth-based assays) or amelioration of the toxicity of a transgene; (ii) immediate validation in other experimental (animal) models before proceeding with target identification. This ensures that compounds only active in yeast can be filtered out as they are less relevant for applications in humans (although the mechanism of action might still shed light on conserved aging processes). When the initial screen was performed in a yeast disease model, the validation should be carried out in a multicellular model for the respective pathology (if available); and (iii) treatment of selected genetic mutant libraries (or any combination thereof) with the validated compound(s) using HIP, HOP, MSP or mutagenesis to identify genetic loci, which modify the effects of the drug (Figure 3). Tardiff *et al.* followed exactly this strategy to identify chemical compounds which ameliorate proteinopathies (Tardiff *et al*., 2012, 2013). Notably, the readout for the third phase of these screens was acute growth impairment upon exposure to the drug, which is usually easier to assess than aging. Therefore, it is worth testing a range of concentrations of the candidate compound: if high substance concentrations cause growth impairment then subsequent target identification can be performed under more stringent conditions, potentially yielding more unambiguous results.

## HIGH-THROUGHPUT METHODS TO MONITOR YEAST AGING

One of the challenges when monitoring aging in any model organism is the lack of dichotomous (e.g. positive or negative) readouts. Most yeast-based screens rely on differences in growth in liquid or solid media, which requires stringent experimental setups such as a clear growth defect of the control condition. However, a compound with anti-aging properties does not necessarily improve cell growth *per se*, but may only compensate the loss of regrowth ability during prolonged aging. There are (at least) three possible strategies to meet the requirements of anti-aging screens: (i) following population survival over time to identify substances which decelerate loss of viability; (ii) linking the effects of drug treatment to more unambiguous phenotypes (e.g. by linking pathway activities to essential genes); or (iii) in the case of target identification procedures, increasing the concentration of the substance of interest until a more distinct phenotype can be observed.

Classic, low-throughput clonogenicity assays, where the ability to form cultures from single cells on solid media is monitored, can be performed in a high-throughput fashion by assessing outgrowth or microcolony formation. The outgrowth capacity (i.e. the ability to divide in fresh liquid media) of an aging culture declines with age and can be monitored by measuring, over several hours, the optical density of a culture aliquot that has been diluted in fresh media. There are commercially available growth curve measuring devices which combine photometric measurements with integrated shaking incubators, facilitating automated readouts. This technique has been used to study the effects of media composition and osmolarity on yeast aging (Murakami *et al.*^2008^), and also in a large-scale screen for genes associated with longevity among the deletion mutants of the yeast knockout collection (Powers *et al.*^2006^). The setup can easily be adapted to compound screens by replacing different yeast strains with different treatments. Microcolony formation is based on the same principle as clonogenicity, but with a faster readout and a high-throughput workflow. Aliquots of an aging yeast culture are spotted onto agar plates and the growth of colonies can be tightly monitored with a BioSpot® Analyzer, which is able to distinguish colonies with a diameter as small as 25 μm (Teng and Hardwick 2013). For anti-aging compound screens, yeast can be treated with compound libraries in 96-, 384-, or even 1536-well plates, and aged in a shaking incubator. At multiple time points during aging, culture aliquots can be taken manually or in an automated fashion using liquid-handling workstations (e.g. QIAGEN BioRobot), then spotted, and microcolonies counted after approximately 18 hours. Compounds that prolong lifespan result in improved formation of microcolonies over time compared to untreated controls. It is of note that both methods measure cell growth and the ability to divide, which does not necessarily correspond to cell viability. Cells may be unable to divide for several reasons, e.g. mutation of growth-relevant genes, but still maintain their viability. Moreover, chemicals that prolong lifespan but also inhibit growth might not be detectable with this approach. For example, cells show reduced growth rates under rapamycin treatment, and when transferred to rapamycin-free media only recover slowly by passive dilution of the drug (Evans, Burgess and Gray 2014). Here, cell vitality as a measure for the physiological capabilities of a cell (Carmona-Gutierrez *et al.*^2018^) can be measured *in situ*, e.g. by flow cytometry-based cell stainings with vital cell dyes (e.g. FUN-1 or alamarBlue®). It should be noted, though, that vital dyes usually detect metabolic activity, for instance, metabolized FUN-1 shifts from green to red fluorescence (Millard *et al.*^1997^). Thus, compounds that attenuate metabolic activity may also reduce FUN-1 conversion and cause a similar phenotype as cell death, although the cell is still viable. Another option is the flow cytometric/photometric quantification of cell death by membrane integrity dyes (e.g. propidium iodide) or cellular ROS levels (e.g. by measuring the ROS-dependent conversion of dihydroethidium to hydroxyethidium and ethidium, respectively), which are good estimators for yeast aging (Pan 2011; Kainz *et al.*^2017^). Importantly, all fluorescence/absorbance-based readouts have to be controlled for background signals that might be caused by the tested compounds. For unbiased anti-aging FCG screens, a combination of these methods will give the most robust results and minimize the occurrence of both false-positive and false-negative hits. Yeast is highly compatible with multi-assay approaches, as it can be aged in multi-well formats, permitting maximal interoperability with automated pipetting robots and high-throughput screen (HTS) readouts.

For high-throughput RLS screens, several single-cell microfluidics methods have been established to overcome the time-demanding manual separation of budded cells (Jo *et al.*^2015^). Large-scale genomic screens have also made use of the so-called ‘old mother cell sorting’, where the surface of young mother cells are labeled with biotin, and after a couple of generations the aged mother cells can be extracted from the culture using streptavidin-coated magnetic beads (Park, Mcvey and Guarente 2002; Sen *et al.*^2015^). In addition, microfluidic approaches to monitor replicative age-dependent intracellular modifications, such as protein abundance and localization changes, have also been developed (Cabrera *et al.*^2017^). However, the adaptability of these methods to pharmacological screens has yet to be established.

## PHARMACOKINETICS AND OFF-TARGET EFFECTS IN YEAST

Yeast has a cell wall that is mostly composed of glucans, mannoproteins and low amounts of chitin, and it has long been believed that the cell wall acts as a potent barrier for molecules with an M_r_ > 700. However, some studies have demonstrated that molecules with significantly higher M_r_ (of up to 400 000) can traverse the cell wall (De Nobel and Barnett 1991). There is little data regarding the influence of the yeast cell wall on the bioavailability of small molecules. As a benchmark, the TORC1 inhibitor rapamycin has an M_r_ of ∼914 and an effective concentration (EC) of 1–10 nM (Powers *et al.*^2006^; Alvers *et al.*^2009^), which is comparable to the EC in human cells (Foster and Toschi 2009). Generally, exponentially growing cells appear to take up externally supplied molecules better than non-dividing cells (De Nobel and Barnett 1991), which should be kept in mind when performing pharmacological screens.

Yeast possesses pleiotropic drug resistance (PDR) efflux pumps which might alter the effective dose of a bioactive compound. To overcome drug efflux, PDR deletion mutants can be employed in pharmacological yeast screens (Griffioen *et al.*^2006^). In some cases, this may increase the sensitivity to drugs by up to 200-fold compared to wild type cells (Rogers *et al.*^2001^). Consequently, a combination of deletion mutants of the paralogs *PDR1* and *PDR3*, which are transcription factors for the pleiotropic drug response, with disruption of the ergosterol biosynthesis gene *ERG6*, which results in increased membrane permeability, greatly enhanced the efficacy of growth-inhibiting compounds in a screen performed at the National Cancer Institute (Simon and Bedalov 2004a). However, the presence of a drug efflux system can also be considered as an advantage of the yeast system, as it models the situation in human cells, which usually comprise functional drug export systems (Chen *et al.*^2016^). Therefore, finding substances which can withstand immediate export is most likely to more efficient in cells with intact drug efflux pumps. In fact yeast has been suggested as a tool for identifying inhibitors of multi drug resistance in cancer cells (Martín-Cordero *et al.*^2013^). In addition, interference with the fungal drug response and cell membrane composition might entail undesired side-effects which could mask normally occurring responses to pharmacological treatment. Nevertheless, when dealing with low amounts of substances, drug efflux mutants are worth considering as models.

Enzymatic modification of xenobiotics, which plays a major role in drug detoxification/degradation in mammals, might only occur to a limited extent in yeast. While yeast does not harbor mixed function type 1–3 cytochrome P450 oxidases (Crešnar and Petrič 2011), which mainly mediate drug metabolization in mammals (Zanger and Schwab 2013), it has a conserved detoxification system by covalent conjugation of xenobiotics to glutathione (Penninckx 2002; Townsend and Tew 2003; Ubiyvovk *et al.*^2006^; Prévéral *et al.*^2009^). However, if specific modification of compounds or activation of prodrugs is required, yeast-expressing human drug metabolization enzymes can be employed. For instance, transgenic yeast-expressing human cytochrome P450 1A2 (CYP1A2) has been used to study the bioactivation and subsequent toxicity of the hepatotoxin Aflatoxin B1 (Guo *et al.*^2005^).

Cell-based screens offer the possibility of estimating toxic off-target effects of the tested compounds. Many substances that are toxic to mammalian cells also kill yeast cells, e.g. the HDAC-inhibitor valproate, the chemotherapy medication paclitaxel, or the anti-cancer compound bleomycin (Almeida *et al.*^2008^). Moreover, yeast-based portable instruments have been developed to estimate genotoxic hazard in environmental monitoring (Knight *et al.*^2004^). Yeast can undergo regulated cell death when exposed to apoptotic stimuli (Madeo, Fröhlich and Fröhlich 1997; Madeo *et al.*^1999^, ^2002^). Interestingly, inter- and intracellular triggers of mammalian apoptosis such as hypochlorite (produced during the oxidative burst in immune cells) or ceramide (the trigger of caspase-independent cell death) also induce apoptosis in yeast in a regulated manner (Carmona-Gutierrez *et al*., 2011, 2013, 2018). Humanized yeast has been used to unravel the adverse side-effects of phenothiazines which are prescribed as antipsychotics (Li *et al.*^2008^): a yeast model expressing the human fatty acid transport protein 2 (FATP2) was treated with a compound library using a fluorescent fatty acid analog as a readout. Phenothiazines efficiently blocked fatty acid uptake, consistent with their metabolic side-effects such as hypertriglyceridemia.

**Figure 4.**
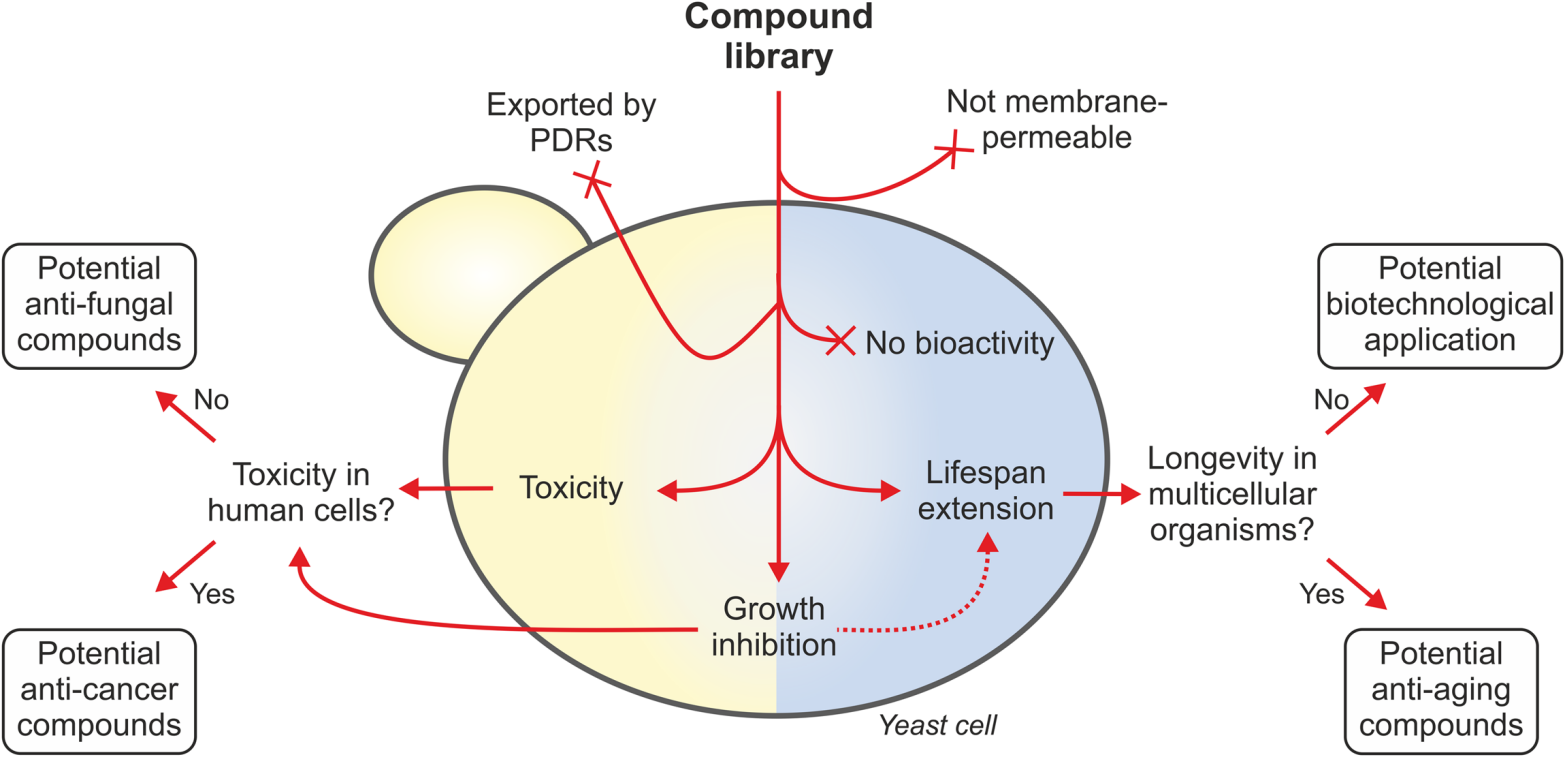
**Versatile transferability of yeast phenotypical screens.** Yeast acts as a cellular sieve for substances which cannot pass the plasma membrane, are exported by pleiotropic drug efflux pumps (PDRs), or generally exhibit no biological activity. Chemicals toxic to yeast can be tested in human cell culture for cytotoxicity. Substances which specifically kill cancer cells are potential chemotherapeutics, while substances with no toxicity might be further tested in pathogenic fungi as potential anti-fungal drugs. Lifespan-extending compounds should be validated in multicellular model organisms (nematodes, flies, mice). Substances which only extend yeast lifespan may be considered for biotechnological applications (e.g. yeast bioreactors) where yeast survival is desired. Chemicals which inhibit growth can be further tested in both directions (e.g. when they inhibit nutrient signaling), but are less suitable for biotechnological use.

In summary, yeast provides a cellular environment that is suited for estimating basic pharmacokinetic properties as well as the off-target effects of a compound of interest (Figure 4). Nevertheless, ECs have to be reconfirmed when transferring the results to other organisms, and a substance that does not exhibit any toxicity in yeast cannot be considered safe for use in other organisms without further testing.

## CONSERVED MECHANISMS OF ANTI-AGING COMPOUNDS IN YEAST

The relevance of a yeast in anti-aging drug discovery is best judged by its successful application both in the mechanistic investigation of aging processes and the identification of strategies to promote longevity. In fact many pharmacological anti-aging interventions follow a conserved mode of action in eukaryotes from yeast to mammals (Table 1). A common target of anti-aging molecules is the regulation of cell growth/proliferation (de Cabo *et al.*^2014^; Jimenez, Ribeiro and Clotet 2015). This is not surprising, since growth signaling has been implicated in aging progression by promoting ROS generation (Weinberger *et al.*^2010^), and genetic interference with cellular growth regulation (e.g. by inhibiting ribosomal function) has repeatedly been shown to promote RLS in yeast (Chiocchetti *et al.*^2007^; Steffen *et al.*^2008^; McCormick *et al.*^2015^). Along similar lines, reducing nutrient signaling, which is most efficiently achieved by caloric restriction (CR), represents an evolutionary conserved means of extending lifespan (Fontana, Partridge and Longo 2010; de Cabo *et al.*^2014^). Therefore, a promising strategy for the identification of anti-aging compounds is to screen for their ability to mimic the effects of CR.

**Table 1.** **Anti-aging compounds from yeast to mammals.** Selected compounds that extend lifespan in yeast and at least two higher eukaryotes.

Substance	Molecular target in yeast	Molecular target in mammals	Anti-aging mechanism	Aging-related clinical trials^a^	Reference
Spermidine	Histone acetyl-transferases	Histone acetyl-transferase EP300	Protein deacetylation, autophagy induction	Observational study (NCT01649960) completed; Phase II clinical trial (NCT03094546) ongoing	(Eisenberg *et al.*^2009^, ^2016^)
Ethanolamine	Phosphatidyl-ethanolamine	Phosphatidyl-ethanolamine	Increase of Atg8-PE levels; autophagy induction	none	(Rockenfeller *et al.*^2015^)
Rapamycin	Tor1	mTORC1	TOR inhibition; autophagy induction; inhibition of translation	Phase I trial (NCT01649960) completed; Phase II trial (NCT02874924) ongoing	(Alvers *et al.*^2009^; Harrison *et al.*^2009^; Prévéral *et al.*^2009^)
Resveratrol	Sir2^b^	SIRT1, TyrRS	Sirtuin activation; protein de-acetylation, autophagy induction	Several beneficial effects in Phase II clinical trials against age-related diseases	(Howitz *et al.*^2003^; Baur *et al.*^2006^; Berman *et al.*^2017^)
Aminoguanidine	unknown	3-deoxyglucosone	Decreased advanced glycation end-products	none	(Oudes *et al.*^1998^; Wang *et al.*^2007^; Kazi *et al.*^2017^)
Metformin	unknown	Respiratory complex I	AMPK activation, autophagy induction	Phase II clinical trial (NCT03309007) ongoing	(Martin-Montalvo *et al.*^2013^; Borklu-Yucel, Eraslan and Ulgen 2015)

^a^trial-identifier at clinicaltrials.gov in parentheses.

^b^direct interaction controversial.

Nutrient availability in yeast is mainly sensed by the target of rapamycin/S6 kinase (TOR/Sch9p), G-protein/protein kinase A (Ras/PKA) and AMP-activated kinase (AMPK, Snf1p in yeast) signaling pathways. Deletion of genes that promote nutrient signaling, such as *RAS2* or *SCH9* (Fabrizio *et al.*^2003^), results in lifespan extension in yeast, which can also be observed when reducing the activity of the corresponding pathways in mice (Yan *et al.*^2007^; Selman *et al.*^2009^). Similarly, treatment with the TORC1 inhibitor rapamycin prolongs yeast lifespan (Powers *et al.*^2006^) and delays aging in mice (Harrison *et al.*^2009^). Low-dose application of rapamycin in healthy adults might decrease senescence markers, but does not improve overall frailty (ClinicalTrial Identifier: NCT01649960; Singh *et al.*^2016^). It is of note that rapamycin treatment leads to immunosuppression (hence its main use against organ rejection), which complicates its use in (healthy) humans (Li, Kim and Blenis 2014). Nevertheless, the mechanistic parallels of rapamycin's mode-of-action across species are stunning: it forms a complex with FK506-binding protein 12 (FKBP12) in mammalian cells, which is necessary for the inhibitory effects on the TOR complex. This ultimately results in the inhibition of translation and the activation of autophagy, an intracellular degradation that recycles macromolecules and organelles (Yin, Pascual and Klionsky 2016). In yeast, rapamycin binds to Fpr1p, an FKBP12 homolog, and then this complex inhibits yeast TORC1, also leading to translational arrest and activation of autophagy (Loewith and Hall 2011). Interestingly, autophagy activation is essential for rapamycin-mediated lifespan extension both in yeast (Alvers *et al.*^2009^) and in fly models (Bjedov *et al.*^2010^). This observation agrees with other studies that indicate the general anti-aging properties of autophagy (Madeo *et al.*^2015^). The AMP-sensing kinase (AMPK) can also induce autophagy by inhibition of TOR and activation of the Atg1 complex upon nutrient scarcity. Activation of AMPK in mammals, for example by supplementation of metformin, which elevates cytosolic AMP levels by inhibiting respiratory complex I, extends lifespan (Martin-Montalvo *et al.*^2013^). In yeast, metformin supplemented in the millimolar range has been shown to counteract aging, which is accompanied by a transcriptional response similar to glucose deprivation (Borklu-Yucel, Eraslan and Ulgen 2015). However, the molecular target in yeast remains to be determined. In humans, metformin is primarily used against type 2 diabetes, since its action on AMPK suppresses gluconeogenesis (Rojas and Gomes 2013). Trials in healthy individuals are ongoing (NCT03309007) or have recently been completed (NCT02432287).

The decisive role in elucidating the regulation and mechanics of the autophagic machinery make yeast an excellent model to screen for autophagy-activating compounds, which may hold great promise for anti-aging interventions. Along these lines, yeast served to identify the anti-aging properties of the natural polyamine spermidine (Eisenberg *et al.*^2009^). Spermidine treatment reduces global histone acetylation and leads to an upregulation of autophagy-related genes. Once again, disruption of autophagy inhibits lifespan extension by spermidine in yeast as well as fly and nematode models. Genetic screens in yeast led to the identification of histone acetyltransferases as possible targets of spermidine, a notion that has been corroborated by experiments in human cell culture (Pietrocola *et al.*^2015^). Oral spermidine supplementation also prolongs the lifespan of mice, improves cardiovascular health in an autophagy-dependent fashion, and consumption of spermidine-rich food inversely correlates with cardiovascular morbidity in humans (NCT03378843; Eisenberg *et al.*^2016^). Other benefits of spermidine treatment, such as improved T-cell response (Puleston *et al.*^2014^), anti-cancer immunosurveillance (Pietrocola *et al.*^2016^) and protection against age-related memory loss (Gupta *et al.*^2013^, ^2016^), make it a potential all-round anti-aging intervention, with autophagy induction as the general mechanism of action (Madeo *et al.*^2018^). A clinical trial built on spermidine's capability to promote cognitive abilities is currently ongoing (NCT03094546).

Autophagy involves the formation of phosphatidylethanolamine (PE)-conjugated Atg8p (the yeast homolog of the autophagy related protein LC3), which is essential for the buildup of double-membrane vesicles that sequester cellular material for degradation in the vacuole (the yeast equivalent of mammalian lysosomes) (Yin, Pascual and Klionsky 2016). Supplementation of the PE precursor ethanolamine induces autophagy and prolongs lifespan in yeast, while blockage of PE biosynthesis has opposite effects (Rockenfeller *et al.*^2015^). Ethanolamine induces autophagy in human cells culture and slows down senescence in mammalian cells and flies, representing yet another molecule that promotes autophagy and lifespan across species. While a phase II clinical trial using orally administered PE in patients with solid tumors is ongoing (NCT02950103), no clinical trials in healthy adults are currently being, or have been, conducted.

The polyphenol resveratrol was identified as a sirtuin-activating compound in a screen using yeast RLS as a readout (Howitz *et al.*^2003^). Sirtuins are NAD^+^-dependent protein deacylases, with a wide variety of substrates and functions (Bheda *et al.*^2016^). Interestingly, resveratrol (promoting protein deacetylation) and spermidine (reducing protein acetylation) have similar effects, including the induction of autophagy in human cells (Morselli *et al.*^2011^). Although the direct interaction of resveratrol with the yeast sirtuin Sir2p has been challenged in later studies (Kaeberlein *et al.*^2005^), the groundwork performed in yeast fostered experiments in higher eukaryotes. In fact there have been other targets identified for resveratrol in human cells, including the tyrosyl transfer-RNA synthetase (TyrRS), which binds resveratrol's tyrosine-like phenolic ring and then translocates to the nucleus to activate stress response pathways (Sajish and Schimmel 2015). It remains to be determined whether this interaction contributes to resveratrol's health-promoting effects and if a similar molecular mimicry occurs in yeast. Resveratrol-mediated health benefits include prolonged lifespan of mice when fed a high-calorie diet (Baur *et al.*^2006^), and improved health parameters in nonhuman primates under an adverse diet (Jimenez-Gomez *et al.*^2013^; Mattison *et al.*^2014^). The therapeutic potential in humans has been corroborated in a series of clinical trials in patients with diverse morbidities (Berman *et al.*^2017^). However, some trials in healthy adults reported improved biomarkers for atherosclerosis (NCT01244360; Agarwal *et al.*^2013^) and inflammation (NCT01492114; Bo *et al.*^2013^), but mixed effects on cerebral blood flow and no effects on cognitive abilities (NCT01640197, NCT01794351; Kennedy *et al.*^2010^). Several compounds that have been discovered as potential anti-aging drugs in yeast screens still await validation in multicellular organisms, including the bile acid lithocholic acid (Goldberg *et al.*^2010^) and the fungal secondary metabolite beauveriolide I (Nakaya *et al.*^2012^). Chemical genetic screens in fission yeast have revealed several pharmacologically accessible cellular processes against aging (Stephan, Franke and Ehrenhofer-Murray 2013). The K^+^/H^+^ exchanger nigericin and Na^+^/H^+^ exchanger monensin prolong fission yeast lifespan by improving vacuolar acidification and reducing vacuolar fragmentation in a vacuolar-ATPase-dependent manner. Interestingly, studies with these compounds in human neuroblastoma cell lines have linked cytosolic acidification to mitochondrial quality control by autophagy (Berezhnov *et al.*^2016^). Here, high concentrations of nigericin led to decreased cytosolic pH and activated mitochondrial-specific autophagy (termed ‘mitophagy’) independent of the canonical PINK1/Parkin signaling cascade. It remains to be determined if this effect can be harnessed to extend lifespan in higher eukaryotes. Vacuolar acidification has been linked to lifespan extension upon methionine restriction in yeast (Ruckenstuhl *et al.*^2014^). It is noteworthy that lysosomal pH in mammalian cells is regulated by mTOR via the lysosomal ATP-sensitive Na^+^ channel lysoNaATP in response to nutrient availability (Cang *et al.*^2013^). Lysosomal acidification/function is an appealing target for pharmacological lifespan extension (Carmona-Gutierrez *et al.*^2016^), and the evolutionary conservation allows screening for compounds in yeast.

The accumulation of advanced glycation end-products (AGEs), which are products of non-enzymatic addition of carbohydrates to other macromolecules, has been linked to the progression of age-related vascular decline, especially in patients with diabetes (Goldin 2006). Glycation inhibitors such as aminoguanidine have been shown to delay replicative senescence in human fibroblasts (Wang *et al.*^2007^) as well as extending lifespan in Drosophila (Oudes *et al.*^1998^). Recently, a study has suggested that aminoguanidine acts in a conserved manner in yeast and prolongs lifespan by reducing AGEs (Kazi *et al.*^2017^).

In summary, yeast is a suitable cellular environment for the identification or mechanistic characterization of anti-aging substances (Figure 4), provided that the results can be reproduced in higher eukaryotes.

## YEAST CHEMOGENOMIC SCREENS AGAINST AGE-RELATED DISEASES

In addition to its application as a model for the aging process, yeast has been increasingly used to study specific human age-related diseases. On the one hand, specific molecular defects might be modeled by genetic manipulation to obtain mechanistic insights into corresponding disorders, e.g. congenital disorders due to defective N-glycosylation or lysosomal storage diseases arising from deficiencies in lysosomal proteins and pathways (Hauptmann *et al.*^2006^; Rajakumar, Munkacsi and Sturley 2017). On the other hand, humanized yeast models heterologously express human (disease-relevant) genes (Menezes *et al.*^2015^; Heinisch and Brandt 2016; Laurent *et al.*^2016^) and can be used to screen compound libraries for specific modulators. Such genes frequently exert cytotoxic effects when expressed in yeast, which enables screening for cell viability or cell growth phenotypes (Figure 3). By using different genetic isoforms of a heterologous gene, yeast can be engineered to facilitate the screening of isoform-/organism-specific therapeutic compounds. Such an approach has been performed, for instance, to identify acetyl-CoA carboxylase 2 (AAC2)-specific inhibitors (Marjanovic *et al.*^2010^).

To some extent such concepts follow the rationale of RCG, as the target or pathway of interest is usually selected *a priori*, but compound treatment is performed in an *in vivo* cell-based fashion. Heterologous expression of disease-related genes, such as neurotoxic proteins, often results in yeast cell death (Shrestha and Megeney 2015; Heinisch and Brandt 2016; Ruetenik *et al.*^2016^; Speldewinde and Grant 2017). Importantly, a common molecular etiology of neurotoxicity is the disturbance of protein quality control. Similar to human cells, yeast is equipped with multiple levels of proteostasis control, including chaperone-mediated protein (re-)folding (Winkler *et al.*^2012^; Mathew and Stirling 2017), endoplasmic reticulum-associated degradation (ERAD) of misfolded proteins (Brodsky and Skach 2011), unfolded protein response (Wu, Ng and Thibault 2014) and proper protein localization through vesicular trafficking (Bonifacino and Glick 2004). For example, expression of the Parkinson's disease (PD)-related protein α-synuclein in yeast leads to disturbance of Rab1p-mediated ER-to-Golgi trafficking and ER stress (Cooper *et al.*^2006^), as well as reduced vacuolar proteolytic function (Aufschnaiter *et al.*^2017^) and results in cytotoxicity, which involves the Golgi-localized Ca^2+^ transporter, Pmr1p, and the mitochondrial nuclease, Nuc1p (yeast homolog of EndoG). Importantly, these mechanisms are conserved in multicellular organisms and neuroblastoma cell lines, respectively, consolidating the use of yeast in elucidating disease mechanisms (Büttner *et al*. 2013a, 2013b). Such yeast strains can be used to screen for substances which ameliorate the phenotype. Indeed, a small molecule screen in yeast using optical density as well as vitality staining with alamarBlue® has been effectively used to discover compounds that ameliorate α-synuclein toxicity by restoring vesicular transport (Fleming *et al.*^2008^; Su *et al.*^2010^). A similar screen performed in a yeast model of Alzheimer's disease (AD) identified a set of small molecules with the capacity to modulate aggregation of amyloid beta, the major pathological hallmark of AD (Amen and Kaganovich 2016; Park *et al.*^2016^). Different yeast-expressing proteotoxic proteins, namely α-synuclein, transactive response DNA-binding protein 43 (TDP-43, involved in frontotemporal dementia) and htt-72Q (the mutant form of huntingtin causing Huntington's disease) revealed 8-hydroxyquinolines (8-OHQs) as potential lead substances against neurodegenerative diseases. The primary readout in this screen was the reconstitution of the transgene-mediated growth defect by treatment with a substance library of ∼190 000 compounds. Remarkably, mechanistic investigations in yeast confirmed that 8-OHQs ameliorated proteotoxicity mainly by intracellular metal chelation, in line with their known ability to form complexes with metal ions (Tardiff *et al.*^2012^). Another hit from this screen that showed promising effects against α-synuclein-mediated toxicity was investigated in detail using a combinatory approach with three different yeast mutant libraries. After a primary screen testing synthetic analogs of the lead substance for their ability to inhibit cell growth at high concentrations, the most promising candidate, N-aryl benzimidazole (NAB), was administered to an overexpression library, a collection of random transposon insertions as well as cells with spontaneous genomic point mutations. The three libraries revealed a network surrounding the ubiquitin ligase, Rsp5p, which is involved in endosomal transport, as the main target for NAB. Importantly, the results could be confirmed in neuronal cell cultures (Tardiff *et al.*^2013^) and neurons derived from humans with PD (Chung *et al.*^2013^). Interestingly, Rsp5p has a C2 calcium-binding domain and, together with the known involvement of Ca^2+^ in α-synuclein-mediated toxicity, this fosters the view of a causal calcium signaling network governing PD, which can be studied in a unicellular organism. The extensive screening strategy preceding the identification of NAB represents the blueprint for the optimal use of yeast as a drug discovery tool. In fact, a comparable approach in neuronal cell culture would not have been feasible, both technically and economically.

Given the conservation of cancer-relevant pathways in yeast, proteins involved in cancer etiology can be expressed in yeast both to study their impact on growth and to identify potential inhibitors (Simon and Bedalov 2004b). In an effort to find novel inhibitors of human sphingosine kinase 1 (SphK1), which phosphorylates sphingosine and shows elevated expression in various cancers, Kashem *et al.* employed a yeast model heterologously expressing SphK1 (Kashem *et al.*^2016^). Expression of this transgene resulted in a lethal phenotype, consistent with previous findings that reported toxicity upon elevation of sphingolipid long-chain base phosphates in yeast (Zhang *et al.*^2001^). Importantly, some of the bioactive compounds found in the yeast model had not been identified in a parallel *in vitro* screen, underlining the relevance of a living cellular screening environment (Kashem *et al.*^2016^). Due to the high grade of conservation in cell cycle genes, pharmacological screens for inhibitors of cyclin-dependent kinases (CDKs), which are essential for cell division and therefore attractive targets for chemotherapy, can be performed in yeast. The yeast *CDC28* gene can be functionally complemented by its human homologs, CDK1 and CDK2, allowing expression of the human proteins in a *cdc28Δ* strain. By using growth inhibition upon compound treatment as a readout, this model has already revealed selective inhibitors of the human CDKs (Mayi *et al.*^2015^).

Mitochondrial function is intimately connected to healthy aging, while mitochondrial disorders are involved in a variety of age-related diseases (Bratic and Larsson 2013). Yeast has been successfully used to identify novel substances that could increase mitochondrial membrane potential and total ATP content, both of which negatively correlate with aging (Montague *et al.*^2014^). A screen performed in cells lacking a subunit of the mitochondrial succinate dehydrogenase (SDH) has been used to screen 200 000 compounds for potential therapeutics against disorders linked to succinate accumulation, such as familial paraganglioma. Interestingly, two of the screen hits were inhibitors of the fungal alcohol dehydrogenase, which in cancer cells plays a similar role in regenerating NAD^+^ as lactate dehydrogenase (LDH). Accordingly, human HEK293 cells treated with siRNA against the SDH component SDHB showed increased susceptibility to the LDH inhibitor oxamate, suggesting LDH as a potential target in paraganglioma therapy (Bancos *et al.*^2013^). In addition to single mitochondrial gene deletions, yeast cells devoid of mitochondrial DNA (ρ^0^ strains) can be used to investigate bioactive compounds in the absence of mitochondria-encoded genes, which distinguishes yeast from other model organisms. Indeed, screens with inhibitors of sphingolipid biosynthesis in the background of ρ^0^ strains have revealed distinct susceptibilities to different compound classes depending on the presence of mitochondrial DNA (Kemmer *et al.*^2009^). When targeting cellular respiration, it should be kept in mind that yeast does not possess a typical respiratory chain complex I, but instead regenerates NAD^+^ by a set of NADH dehydrogenases located on both sides of the inner mitochondrial membrane (Lasserre *et al.*^2015^).

## THE ADDED VALUE OF YEAST-BASED SCREENS

In the realm of model organisms, yeast has unique properties that allow transferring the results of a single screen to different applications. First, yeast is a valid model for human aging, and compounds that prolong yeast lifespan are good candidates for anti-aging interventions in humans. Important selection criteria for anti-aging compounds include improved lifespan (CLS or RLS or both) or modulation of aging-related pathways (e.g. nutrient/growth signaling, autophagy, proteostasis or ROS generation), drug bioavailability (in yeast mainly influenced by membrane permeability or drug export), no or low toxicity (tested by assessing cell viability/vitality). Lifespan-extending compounds should be validated in established multicellular model organisms such as nematodes, flies or mice (Buffenstein, Edrey and Larsen 2008). Thereby, appropriate control experiments for each model (e.g. food consumption, drug bioavailability) should be included, especially when the compound is administered *ad libitum* via food or drinking water. To facilitate the translational use of compounds, pharmacokinetic properties should be determined for each compound *in vivo* or pre-estimated based on the chemical properties using online tools such as SwissADME (http://www.swissadme.ch). Lifespan-extending hits from yeast screens that cannot be validated in other organisms may have biotechnological applications in cases where yeast aging has adverse effects, such as beer fermentation (Brányik *et al.*^2008^). Here, the underlying mechanism and ways to reproduce the effects by genetic means rather than the substance itself are of interest, as the addition of uncharacterized compounds to bioreactors is usually not preferred. Second, yeast can be used as a model for fast-growing mitotic cells to identify anti-cancer agents (Simon and Bedalov 2004b). Compounds toxic to yeast can be tested in human cell culture (both in malignant and non-malignant cell lines) for cytotoxicity. Substances that specifically kill cancer cells are potential anti-cancer agents. Notably, substances that are cytotoxic in yeast, or inhibit yeast growth but do not show any activity in human cancer cell lines, might be considered as potential anti-fungal compounds, provided that they do not exhibit any toxic effects in non-malignant cell lines and mice. Indeed, due to its close relatedness to fungal pathogens like *Candida* (Berman and Sudbery 2002), *S. cerevisiae* is a widely-used model for the mechanistic examination of antifungal drugs (Tebbets *et al.*^2012^; Roberts, Miller and Atkinson 2017; Serratore *et al.*^2017^). Thus, given an appropriate readout (e.g. lifespan or cell stress), results from a single screen can be used for a variety of translational studies (Figure 4).

## CONCLUDING REMARKS

Yeast has emerged as one of the most versatile organisms both in basic research and applied science. The small and comparatively well-characterized genome makes yeast a prime model to study complex cellular processes in a simple environment. The fruitful translation of findings from yeast to higher eukaryotes has helped to understand aging processes in humans, including the mode of action of anti-aging molecules like rapamycin, spermidine or resveratrol. Multiple pharmacological screens have used yeast for initial drug discovery, and often the screening procedure would have been unfeasible in other experimental models. Yet yeast has long been overlooked as a tool to screen for anti-aging compounds. The growing number of available tools and methods to assess lifespan in a high-throughput fashion further paves the way for yeast-based anti-aging screens. In addition, yeast viability is a powerful readout that allows translational use of bioactive compounds in research fields besides aging, based on the hypothesis that compounds that reduce viability might be applicable against cancer or fungal infections. In this regard, data from completed studies could be revisited and mined for potential bioactive substances. However, data obtained in yeast should not be over-interpreted unduly, and when aiming for applications in humans, validation of compounds in multicellular organisms is a *sine qua non*. Nevertheless, the potential of yeast to unveil novel pharmacological interventions against aging is far-reaching and we are sure that it will continue to contribute substantially to drug discovery in the field.

## FUNDING

This work was supported by the Austrian Science Fund FWF [SFB-LIPOTOX F3007, F3012, P23490-B20, P24381, P27893, P29203, P29262, W1226]; the European Commission [APOSYS]; the Austrian Federal Ministry of Science, Research and Economy [BMWFW-80.109/0001-WF/V/3b/2015], BioTechMed-Graz [EPIAge], and the University of Graz [Unkonventionelle Forschung]. We acknowledge support from NAWI Graz.

***Conflict of interest.*** None declared.
